# Author Correction: Characterization of crAss-like phage isolates highlights Crassvirales genetic heterogeneity and worldwide distribution

**DOI:** 10.1038/s41467-023-42062-3

**Published:** 2023-10-04

**Authors:** María Dolores Ramos-Barbero, Clara Gómez-Gómez, Laura Sala-Comorera, Lorena Rodríguez-Rubio, Sara Morales-Cortes, Elena Mendoza-Barberá, Gloria Vique, Daniel Toribio-Avedillo, Anicet R. Blanch, Elisenda Ballesté, Cristina Garcia-Aljaro, Maite Muniesa

**Affiliations:** Departament de Genètica, Microbiologia i Estadística, Universitat de Barcelona, Diagonal 643. Annex. Floor 0, E-08028 Barcelona, Spain

**Keywords:** Bacteriophages, Microbiome, Water microbiology

Correction to: *Nature Communications* 10.1038/s41467-023-40098-z, published online 18 July 2023

In this article, the funding from ‘the ICREA Academia 2022 program’ was omitted. The original article has been corrected.

