# Supplementary material for: Characterization of crAss-like phage isolates highlights Crassvirales genetic heterogeneity and worldwide distribution
Source: Nat Commun. 2023 Jul 18;14:4295. doi: 10.1038/s41467-023-40098-z (PMC10354031; doi:10.1038/s41467-023-40098-z)
Supplement: Supplementary file 1 — Supplementary Information [file 41467_2023_40098_MOESM1_ESM.pdf]

SUPPLEMENTARY INFORMATION

Characterization of crAss-like phage isolates highlights Crassvirales genetic heterogeneity and worldwide distribution

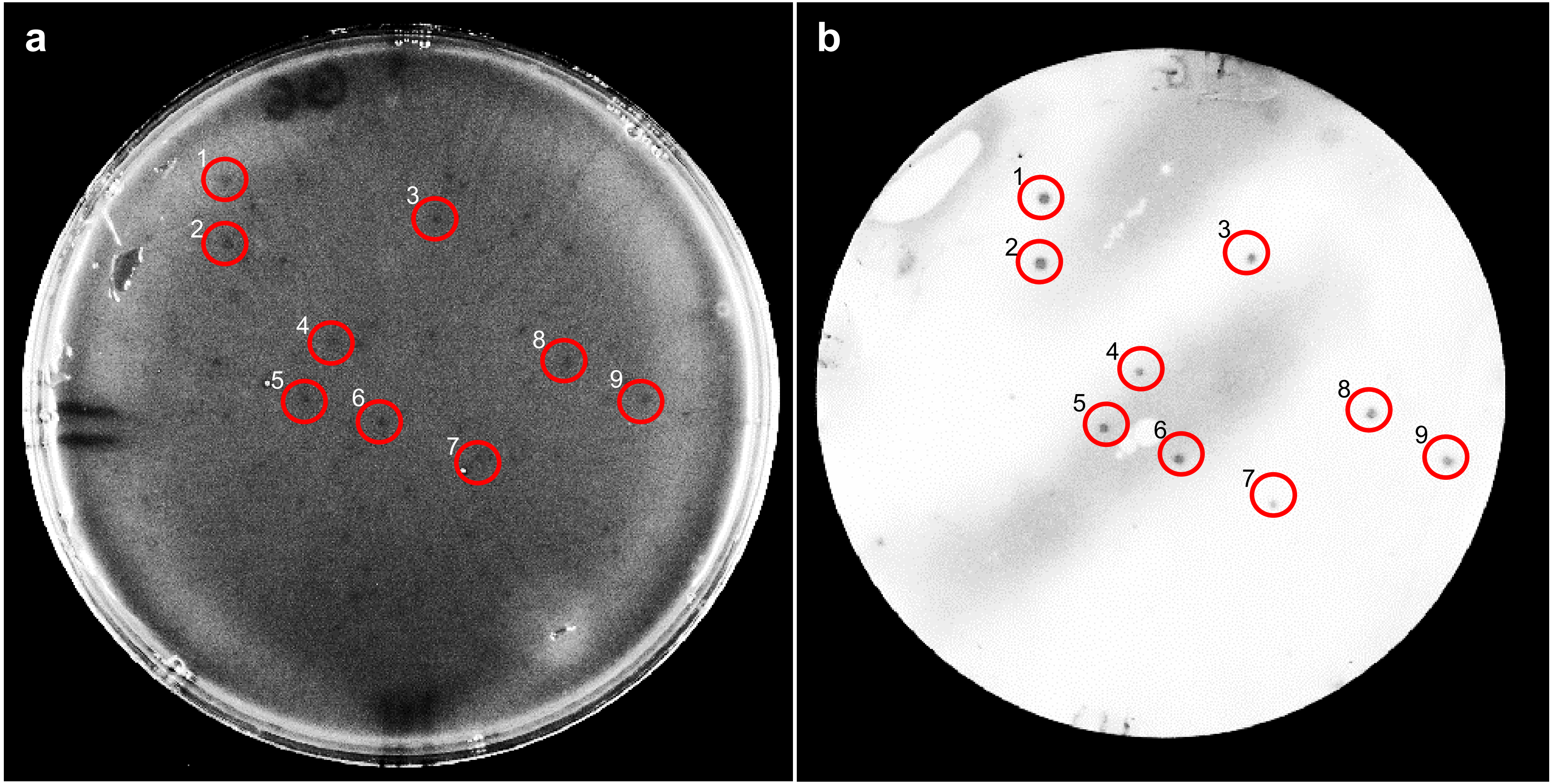

Supplementary Fig. 1. Detection of crAssphage lysis plaques. Representative morphology of the plaques produced by the crAssBcn phages in *B. intestinalis* and plaque blot hybridization. (a) Plaque enumeration by the double agar layer method of phage ΦCrAssBcn6 infecting *B. intestinalis* from a wastewater sample (WWTP Gava 1). (b) Plaque blot hybridization of phages transferred from (a) using the CrAss1-ORF46 probe.

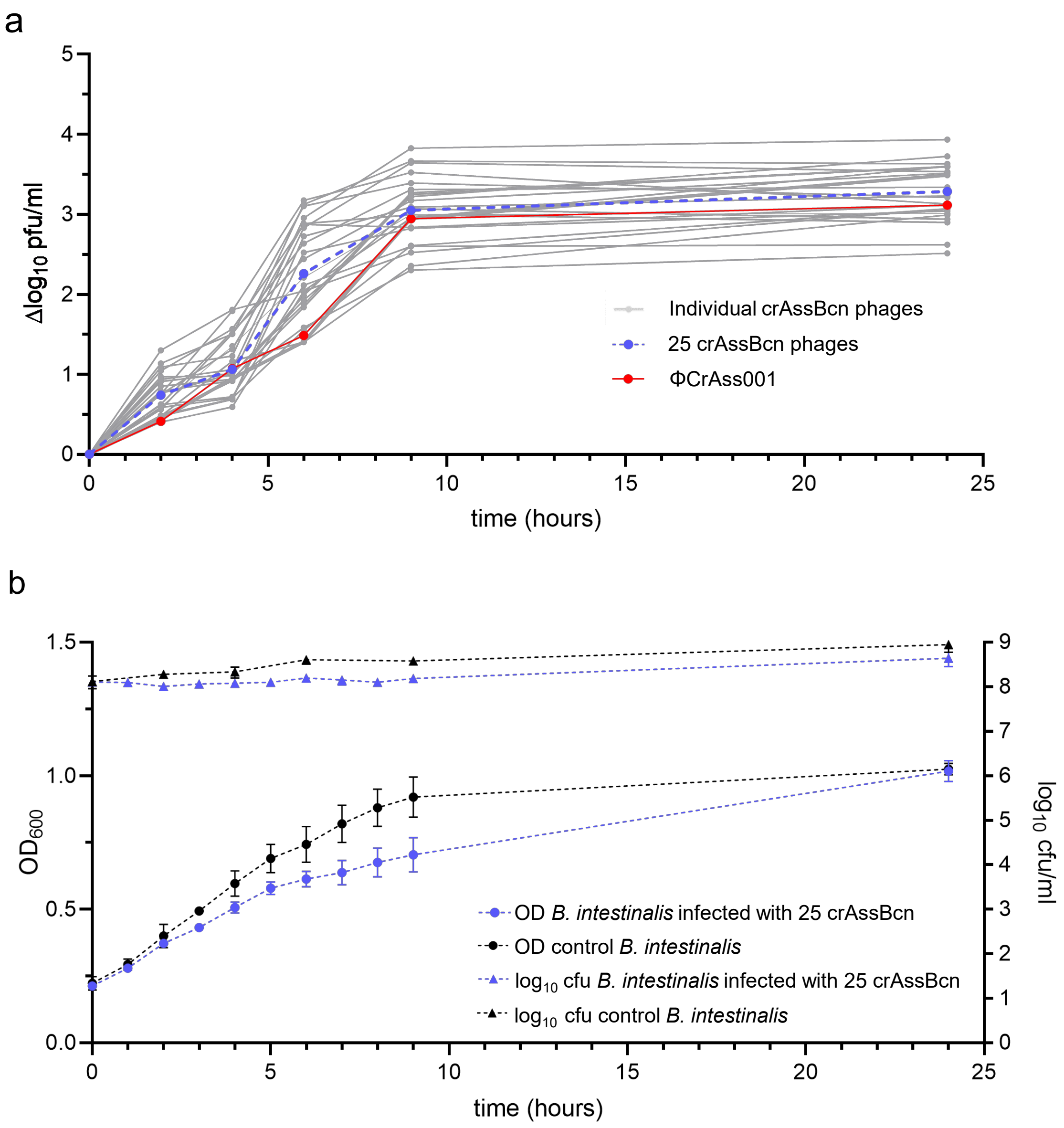

Supplementary Fig. 2. Infectivity dynamics of crAssBcn phages. (a) Increase in the number of infectious phage particles measured during 24 h by the double agar layer method and expressed in $\log_{10}$ pfu/ml. Values in the grey lines (crAssBcn phages) and red (ΦCrAss001) are the average of three replicates performed with each individual phage. Blue dotted line corresponds to the median of the 25 crAssBcn phages (b) Growth of the host strain *B. intestinalis* plotted on the left Y-axis was measured by the increase in optical density (OD) at 600 nm and values are presented with circles. Growth of the host strain *B. intestinalis* plotted on the right Y-axis was measured by colony counts (cfu/ml) and values are presented with triangles. Each value corresponds to the median of seven bacterial cultures uninfected (black) or infected with the 25 crAssBcn phages (blue). Source data are provided as a Source Data file.

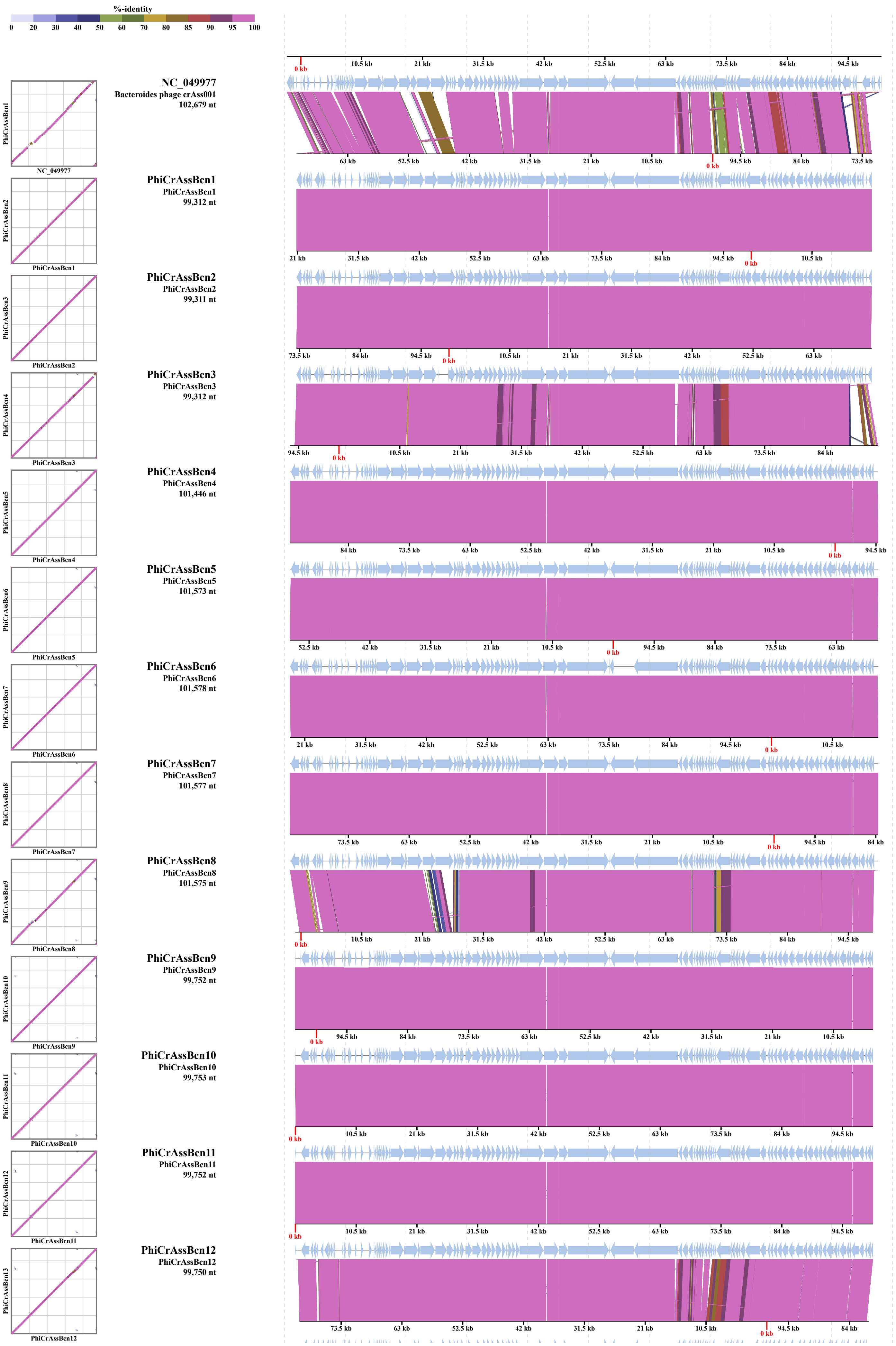
%-identity
0 20 30 40 50 60 70 80 85 90 95 100
NC_049977
Bacteroides phage crAss001
102,679 nt
PhiCrAssBcn1
PhiCrAssBcn1
99,312 nt
PhiCrAssBcn2
PhiCrAssBcn2
99,311 nt
PhiCrAssBcn3
PhiCrAssBcn3
99,312 nt
PhiCrAssBcn4
PhiCrAssBcn4
101,446 nt
PhiCrAssBcn5
PhiCrAssBcn5
101,573 nt
PhiCrAssBcn6
PhiCrAssBcn6
101,578 nt
PhiCrAssBcn7
PhiCrAssBcn7
101,577 nt
PhiCrAssBcn8
PhiCrAssBcn8
101,575 nt
PhiCrAssBcn9
PhiCrAssBcn9
99,752 nt
PhiCrAssBcn10
PhiCrAssBcn10
99,753 nt
PhiCrAssBcn11
PhiCrAssBcn11
99,752 nt
PhiCrAssBcn12
PhiCrAssBcn12
99,750 nt
PhiCrAssBcn13
0 kb
10.5 kb
21 kb
31.5 kb
42 kb
52.5 kb
63 kb
73.5 kb
84 kb
94.5 kb

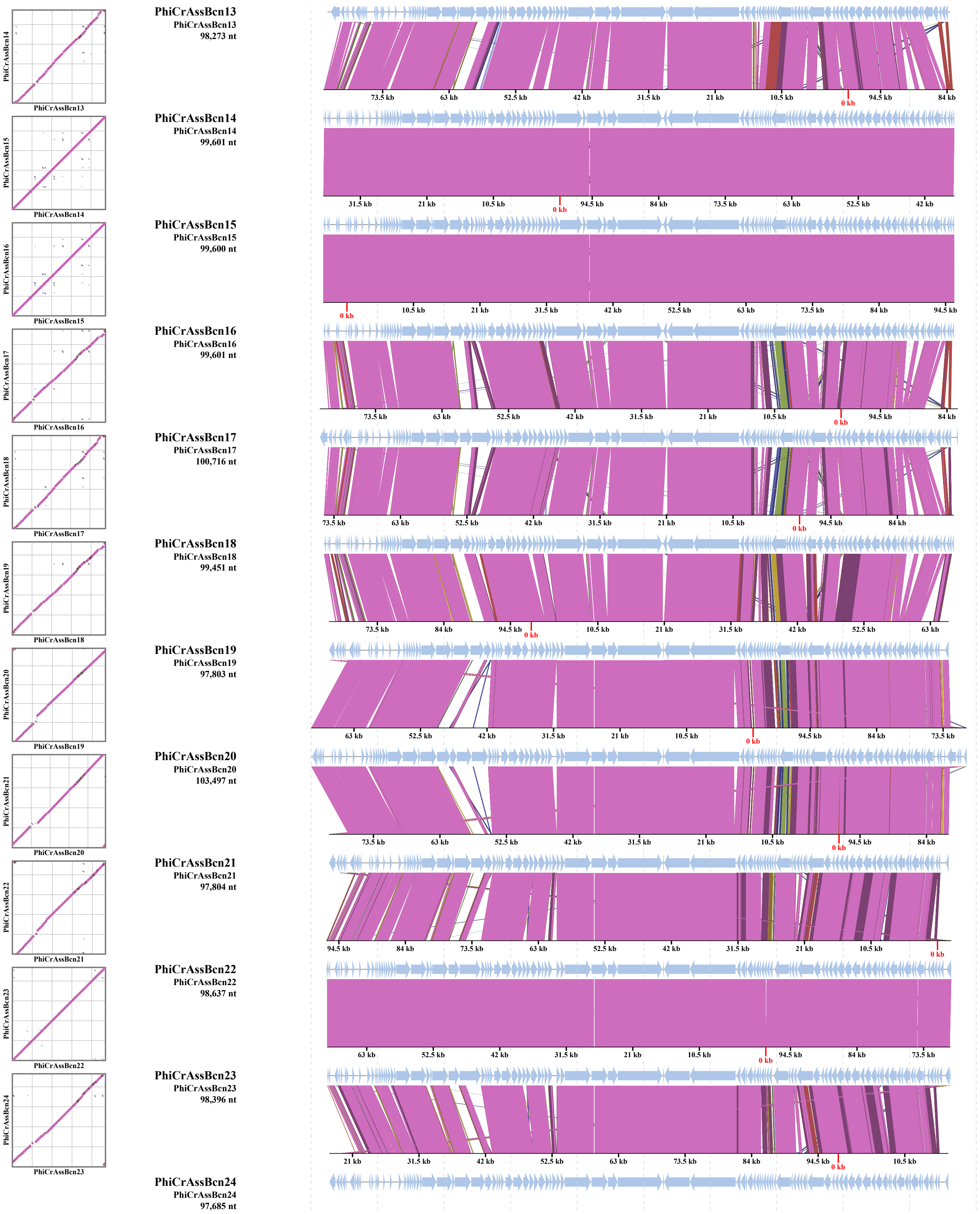

Supplementary Figure 3. Multiple genome alignment of the 24 CrAssBcn phages .CDS alignment of the 24 complete genomes and ΦCrAss001. Left charts show pairwise alignment of two consecutive genomes. Light blue arrows in the genetic maps (right side) indicate the ORFs in each genome. The color of the bands between the genetic maps shows the percentage of identity between each sequence as indicated in the legend at the top of the figure.

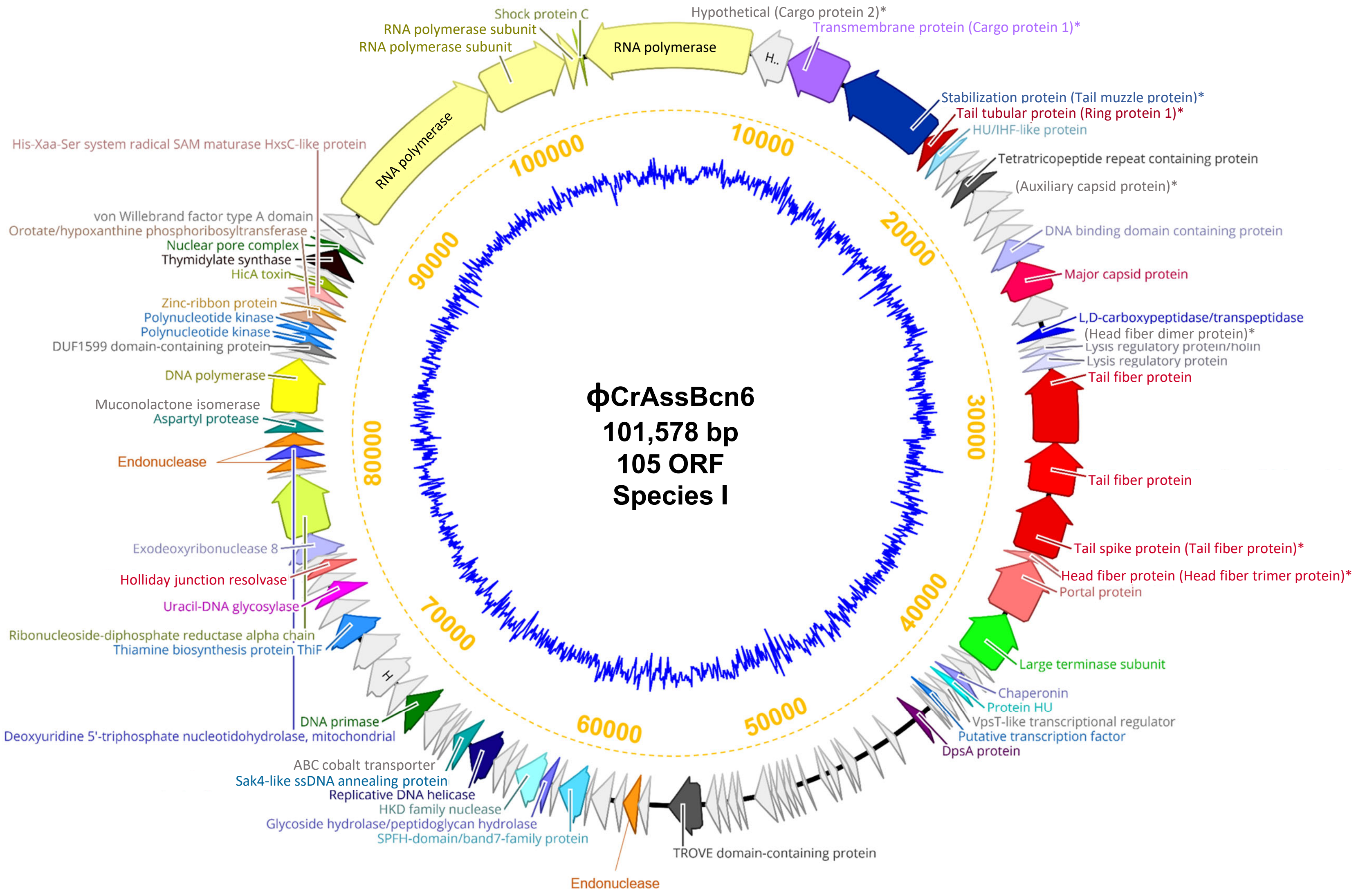

Supplementary Fig. 4. Circular genomic map of ΦCrAssBcn6. Genomic map of the representative phage of the most abundant species (group I) among the crAssBcn phages. The map was generated using Geneious Prime 2022.0.2. Different colored arrows show the predicted ORFs: yellow, with replicative functions; red, structural genes; orange, endonucleases; and grey, hypothetical proteins. The orange dashed ring indicates the genome position, and the inner blue ring displays the variations in the % Guanine/Cytosine. (*) In brackets the ORFs with new annotation reported in Bayfield *et al*., 2022.

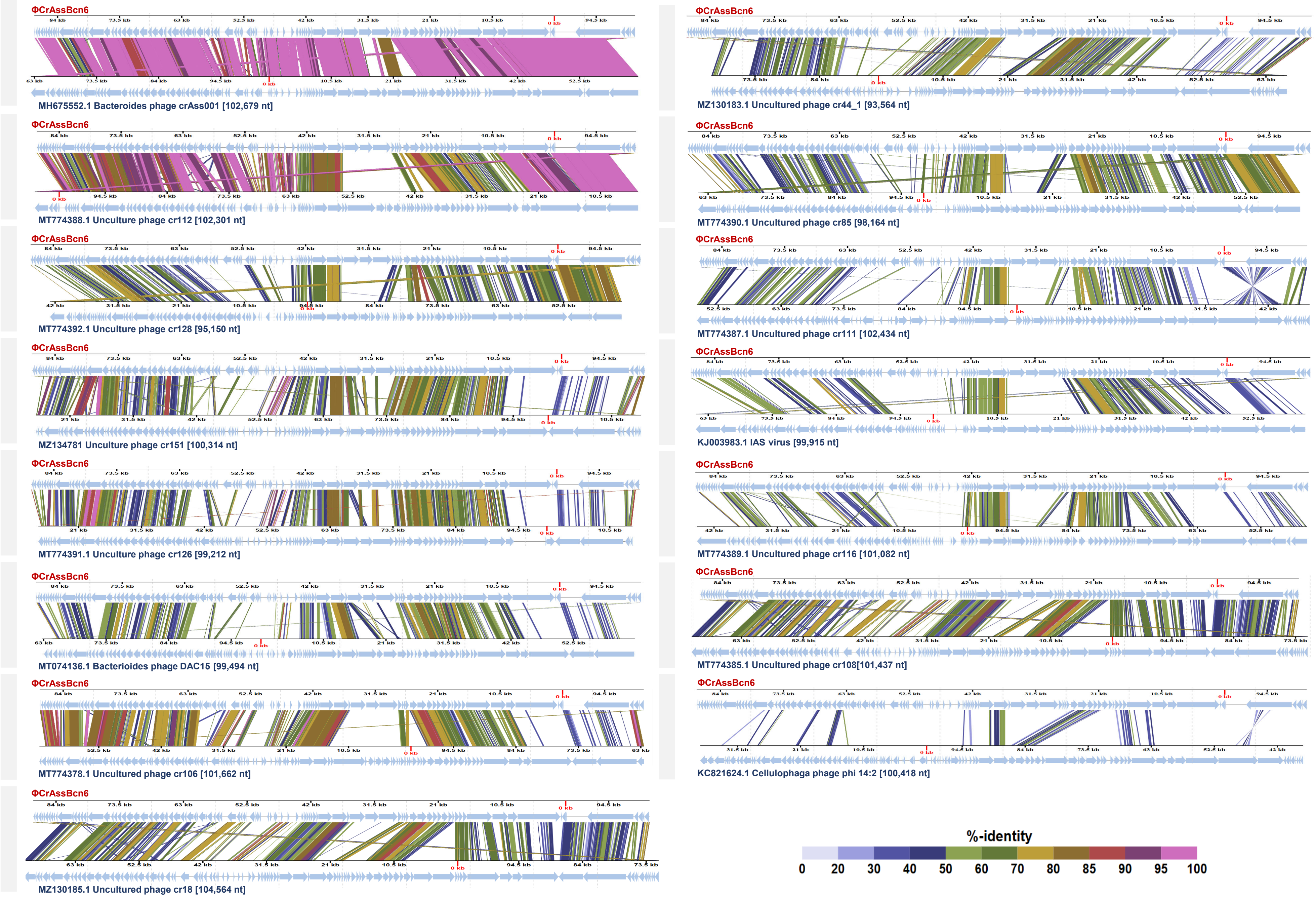

Supplementary Figure 5. Genetic identity of ΦCrAssBcn6 with phages of *Steigviridae* family. CDS alignment of the genome of phage ΦCrAssBcn6 with each one of the 15 crAss-like phage genomes assigned as species of the *Steigviridae* family. Light blue arrows indicate the ORFs in each genome. The color of the bands between the seven genetic maps shows the percentage of identity between each sequence as indicated in the legend at the bottom of the figure.

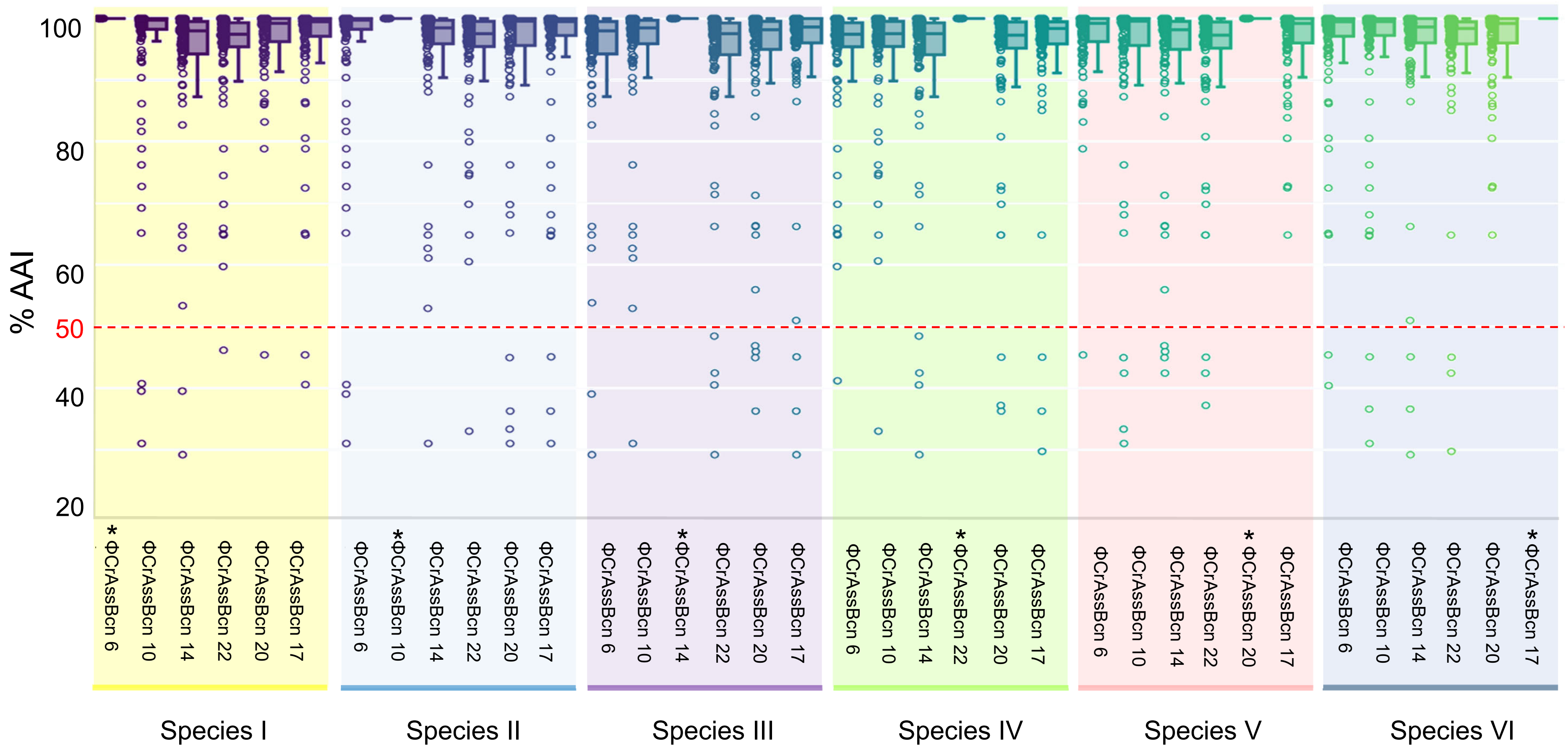

Supplementary Fig. 6. Comparison of the ORFs performed by reciprocal best match of the crAssBcn phages. The boxplot shows the percentage of amino acid identity (% AAI) of all ORFs comparing the representative crAssBcn phage of each species (marked with an *) with those of the other seven species (I-VI). (Nº ORFs for species I = 106, N for species II = 104, N for species III = 114 and N for species IV, V and VI = 105). The ORFs in the boxplots and upper circles have the highest % AAI and therefore the least variability among the phages. Central line of each boxplot represents the average value of the positive samples, the upper box include samples whose values are within the 75th percentile, and those in the lower white boxes are within the 25th percentile. The ORFs with higher variability correspond to the circles below 50 % (dotted red line). Source data are provided in Supplementary Data 2.

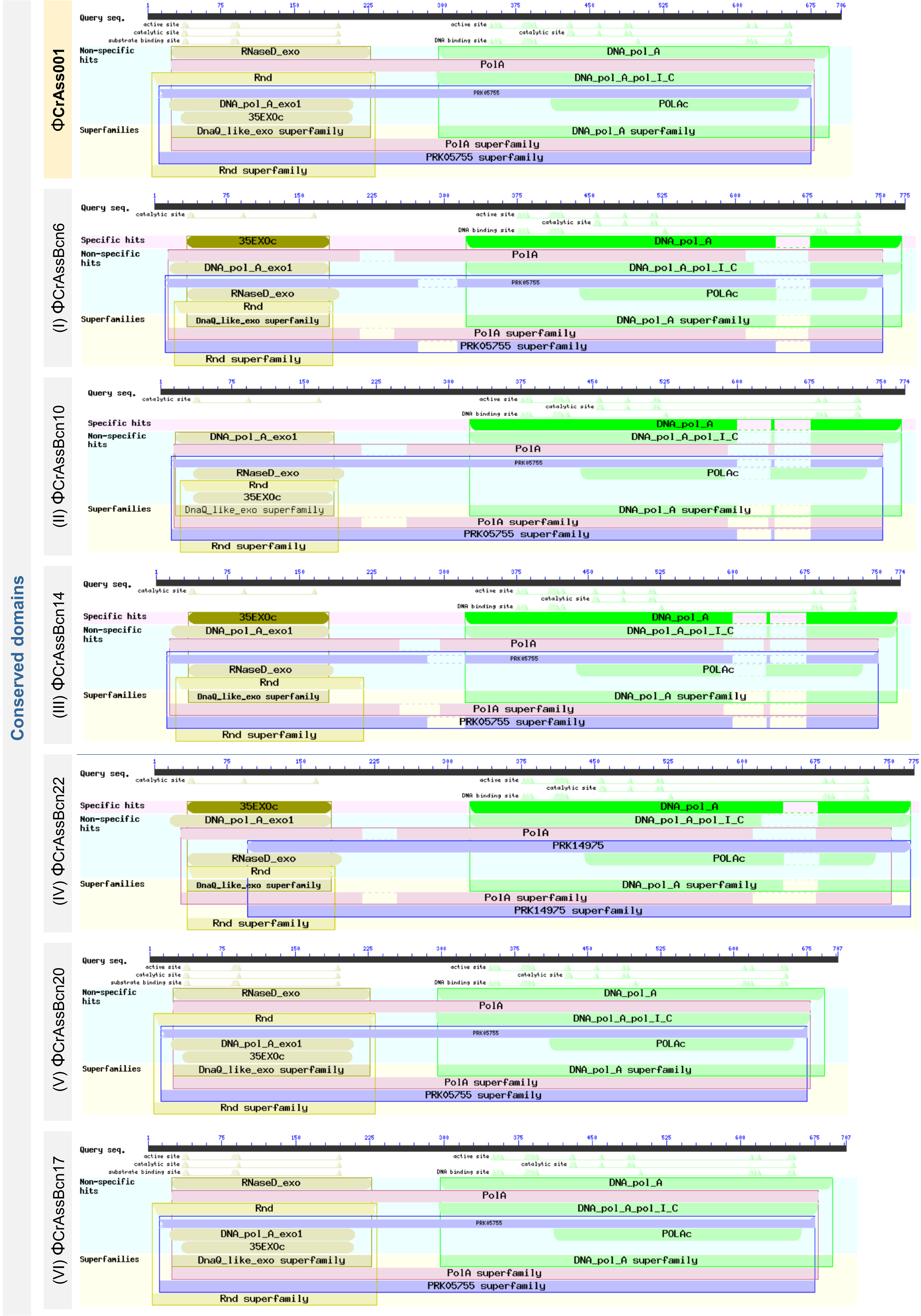

Supplementary Fig. 7. Conserved domains of the polymerase A of crAssBcn phages. Conserved domains of the product encoded in the ORF corresponding to polymerase A of each of the six crAssBcn phages representative of species I-VI. From top to bottom each chart shows first the length of the protein. Small triangles indicate the aminoacids involved in conserved active, catalytic and DNA binding sites of each domain. Colored bars show the closest hits found in the CDD for each domain are presented, these can be specific hits (with a high confident association) or non-specific and the superfamily to which the highest-ranking hit belongs.

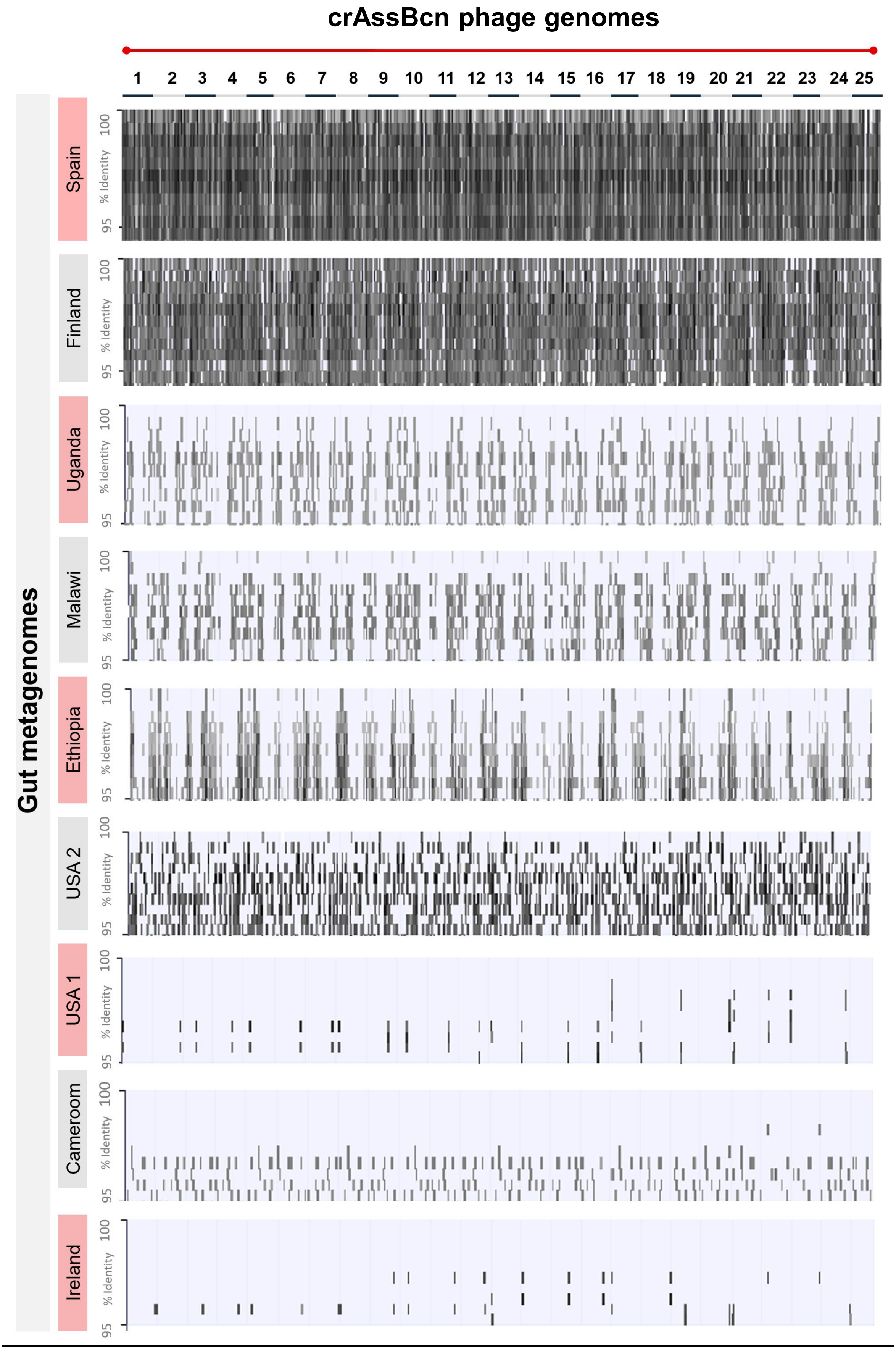

Supplementary Fig. 8. Recruitment plots of crAssBcn phages mapping in gut viral communities from different countries. This figure showcases the results of a BLASTn search of metagenomic short reads against crAssBcn phage genomes (X-axis). The upper panel shows the crAssBcn phage genomes. Bars lower than the average represent regions with fewer mapped reads. The panel for the metagenome of each country represents the reads recruited for each phage, where the X-axis shows mapped reads and the Y-axis the percent identity (from 95 to 100 % of identity).

Supplementary Table 1- Evaluation of crAss-like phages in wastewater samples and after three consecutive propagations in *B. intestinalis* enrichment cultures. Values are expressed in Ct and in brackets are the estimated number of GC/ml, that corresponds to the number of CrAss-like phage viral particles.

WWTP	Ct (GC/ml)				CrAss-like phage*	Isolate
	Initial sample	1rst propagation	2nd propagation	3rd propagation	pfu/ml	
Besos 1	28.96	32.42	17.95	n.a	$6.0 \cdot 10^{7}$	ΦCrAssBcn1
	(10^{4})	(10^{3})	(10^{8})			
Besos 2	31.95	24.96	13.22	n.a	$2.0\ 10^{8}$	ΦCrAssBcn2
	(10^{4})	(10^{6})	(10^{9})			
Besos 3	30.76	26.74	14.09	n.a	$1.0\ 10^{9}$	ΦCrAssBcn3
	(10^{4})	(10^{5})	(10^{9})			
Gava 1	29.18	25.16	14.78	13.28	$3.9 \cdot 10^{8}$	ΦCrAssBcn4
	(10^{4})	(10^{6})	(10^{8})	(10^{9})		ΦCrAssBcn5
						ΦCrAssBcn6
						ΦCrAssBcn7
						ΦCrAssBcn8
Gava 2	28.37	30.84	33.81	33.22	n.a	-
	(10^{5})	(10^{4})	(10^{3})	(10^{3})		
Igualada 1	30.44	24.98	12.03	13.60	$4.4 \cdot 10^{9}$	ΦCrAssBcn9
	(10^{4})	(10^{6})	(10^{9})	(10^{9})		ΦCrAssBcn10
						ΦCrAssBcn11
						ΦCrAssBcn12
						ΦCrAssBcn13
Igualada 2	30.03	34.16	34.09	34.59	n.a	-
	(10^{4})	(10^{3})	(10^{3})	(10^{3})		
Manresa 1	31.24	25.81	10.70	14.21	$1.4 \cdot 10^{9}$	ΦCrAssBcn14
	(10^{4})	(10^{5})	(10^{10})	(10^{9})		ΦCrAssBcn15
						ΦCrAssBcn16
						ΦCrAssBcn17
						ΦCrAssBcn18
Manresa 2	30.32	33.40	33.45	31.62	n.a	-
	(10^{4})	(10^{3})	(10^{3})	(10^{4})		
Prat de	31.18	30.02	13.92	14.28	$2.8 \cdot 10^{9}$	ΦCrAssBcn19
Llobregat 1	(10^{4})	(10^{4})	(10^{9})	(10^{9})		ΦCrAssBcn20
						ΦCrAssBcn21
Prat de	32.98	21.02	13.89	13.54	$8.0 \cdot 10^{8}$	ΦCrAssBcn22
Llobregat 2	(10^{3})	(10^{7})	(10^{9})	(10^{9})		ΦCrAssBcn23
Prat de	32.98	23.31	14.72	n.a	$1.8 \cdot 10^{8}$	ΦCrAssBcn24
Llobregat 3	(10^{3})	(10^{6})	(10^{8})			ΦCrAssBcn25

*Detected by plaque blot hybridization on plates from the last propagation step.
n.a: not analyzed
WWTP: wastewater treatment plant

Supplementary Table 2.- Bacterial strains used in this study.

Bacteria	Strain	Characteristics	Reference/source
Bacteroides fragilis	RYC2056	Clinical isolate	[1]
Bacteroides fragilis	HSP40	Clinical isolate	[2]
Bacteroides sp.	GB105	Municipal wastewater isolate. UK	Our collection
Bacteroides fragilis	GB124	Municipal wastewater isolate. UK	[3]
Bacteroides fragilis	GB307	Municipal wastewater isolate. UK	[4]
Bacteroides fragilis	GB331	Municipal wastewater isolate. UK	[4]
Bacteroides fragilis	GB135	Municipal wastewater isolate. UK	[4]
Bacteroides fragilis	NCTC 9343	Appendix abscess isolate	[5]
Bacteroides fragilis	3156	Abscess isolate	[6]
Bacteroides fragilis	DSM 2079	Human faeces isolate	[7]
Bacteroides fragilis	C13525	Clinical isolate	[2]
Bacteroides fragilis	ATCC 23745	Pleural fluid isolate	[8]
Bacteroides fragilis	C17898	Clinical isolate	[2]
Bacteroides fragilis	C350	Clinical isolate	Our collection
Bacteroides fragilis	C13182	Clinical isolate	[2]
Bacteroides fragilis	D1416	Clinical isolate	[2]
Bacteroides fragilis	638R C17898	Plasmid-free spontaneous rif^R mutant	[9]
Bacteroides fragilis	SA208	Municipal wastewater isolate. Barcelona	Our collection
Bacteroides fragilis	GA18	Municipal wastewater isolate. Barcelona	Our collection
Bacteroides fragilis	Ø7	Municipal wastewater isolate. Tarragona	Our collection
Bacteroides fragilis	PL122	Municipal wastewater isolate. Tarragona	[10]
Bacteroides fragilis	01-HSP	Clinical isolate	Provided by F. Navarro
Bacteroides fragilis	02-HSP	Clinical isolate	Provided by F. Navarro
Bacteroides fragilis	06-HSP	Clinical isolate	Provided by F. Navarro
Bacteroides fragilis	07-HSP	Clinical isolate	Provided by F. Navarro
Bacteroides fragilis	08-HSP	Clinical isolate	Provided by F. Navarro
Bacteroides fragilis	09-HSP	Clinical isolate	Provided by F. Navarro
Bacteroides fragilis	11-HSP	Clinical isolate	Provided by F. Navarro
Bacteroides fragilis	*ΔmpiM44*	Constitutively expresses PSA	Provided by L.E. Comstock
Bacteroides fragilis	*Δtsr19M5*	Expresses extracellular polysaccharide EPS	Provided by L.E. Comstock
Bacteroides fragilis	*Δtsr15M8*	Constitutively expresses an external protein operon	Provided by L.E. Comstock

Bacteroides fragilis	*Δtsr15M4*	Does not express surface proteins that are constitutively expressed in the mutant *Δtsr15M8* Mutant of strain NCTC 9343	Provided by L.E. Comstock
Bacteroides fragilis	*ΔmpiM44ΔupaZΔupeZ*	Constitutively expresses PSA, PSE and PSC. Mutant of strain NCTC 9343	Provided by L.E. Comstock
Bacteroides fragilis	*ΔPSA*	Overexpresses PSA. Mutant of strain NCTC 9343	[11]
Bacteroides fragilis	*ΔungD1ΔungD2*	Expresses PSH. Mutant of strain NCTC 9343	Provided by L.E. Comstock
Bacteroides fragilis	*ΔPSC*	Overexpresses PSC. Mutant of strain NCTC 9343	Provided by L.E. Comstock
Bacteroides fragilis	*Δtsr19M3*	Does not express EPS. Mutant of strain NCTC 9343	Provided by L.E. Comstock
Bacteroides thetaiotaomicron	GA17	Municipal wastewater isolate. Barcelona	[12]
Bacteroides thetaiotaomicron	ATCC 29741	Perforated appendix isolate	[13]
Bacteroides thetaiotaomicron	HB13	Municipal wastewater isolate. Colombia	[12]
Bacteroides thetaiotaomicron	SA1610	Municipal wastewater isolate. Barcelona	[12]
Bacteroides thetaiotaomicron	04-HSP	Clinical isolate	Provided by F. Navarro
Parabacteroides distasonis	ATCC 8503	Human faeces isolate	[14]
Bacteroides ovatus	03-HSP	Clinical isolate	Provided by F. Navarro
Bacteroides ovatus	05-HSP	Clinical isolate	Provided by F. Navarro
Bacteroides ovatus	10-HSP	Clinical isolate	Provided by F. Navarro
Bacteroides intestinalis	DSM 108646	Human faeces isolate	[15]

Supplementary Table 3.- Genome characteristics of the 25 crAssBcn phages in this study, Species group and GenBank accession numbers

Phage	Length (bp)	Complete genome	% GC	Nº ORFs	Species group	GenBank Accession number	Hyperlink
ΦCrAssBcn1	99312	Yes	35.07	102	1	OQ221536	https://www.ncbi.nlm.nih.gov/nuccore/OQ221536
ΦCrAssBcn2	99311	Yes	35.07	102	1	OQ221537	https://www.ncbi.nlm.nih.gov/nuccore/OQ221537
ΦCrAssBcn3	99312	Yes	35.07	102	1	OQ221538	https://www.ncbi.nlm.nih.gov/nuccore/OQ221538
ΦCrAssBcn4	101446	Yes	35.18	103	1	OQ221539	https://www.ncbi.nlm.nih.gov/nuccore/OQ221539
ΦCrAssBcn5	101573	Yes	35.18	105	1	OQ221540	https://www.ncbi.nlm.nih.gov/nuccore/OQ221540
ΦCrAssBcn6*	101578	Yes	35.18	105	1	OQ221541	https://www.ncbi.nlm.nih.gov/nuccore/OQ221541
ΦCrAssBcn7	101577	Yes	35.18	105	1	OQ221542	https://www.ncbi.nlm.nih.gov/nuccore/OQ221542
ΦCrAssBcn8	101575	Yes	35.17	105	1	OQ221543	https://www.ncbi.nlm.nih.gov/nuccore/OQ221543
ΦCrAssBcn9	99752	Yes	35.08	104	2	OQ221544	https://www.ncbi.nlm.nih.gov/nuccore/OQ221544
ΦCrAssBcn10*	99753	Yes	35.08	104	2	OQ221545	https://www.ncbi.nlm.nih.gov/nuccore/OQ221545
ΦCrAssBcn11	99752	Yes	35.08	104	2	OQ221546	https://www.ncbi.nlm.nih.gov/nuccore/OQ221546
ΦCrAssBcn12	99750	Yes	35.08	103	2	OQ221547	https://www.ncbi.nlm.nih.gov/nuccore/OQ221547
ΦCrAssBcn13	98273	Yes	35.19	101	2	OQ221548	https://www.ncbi.nlm.nih.gov/nuccore/OQ221548
ΦCrAssBcn14*	99601	Yes	34.66	114	3	OQ221549	https://www.ncbi.nlm.nih.gov/nuccore/OQ221549
ΦCrAssBcn15	99600	Yes	34.67	114	3	OQ221550	https://www.ncbi.nlm.nih.gov/nuccore/OQ221550
ΦCrAssBcn16	99601	Yes	34.66	114	3	OQ221551	https://www.ncbi.nlm.nih.gov/nuccore/OQ221551
ΦCrAssBcn17*	100716	Yes	35.01	104	6	OQ221552	https://www.ncbi.nlm.nih.gov/nuccore/OQ221552
ΦCrAssBcn18	99451	Yes	34.66	110	3	OQ221553	https://www.ncbi.nlm.nih.gov/nuccore/OQ221553
ΦCrAssBcn19	97803	Yes	35.18	104	1	OQ221554	https://www.ncbi.nlm.nih.gov/nuccore/OQ221554
ΦCrAssBcn20*	103497	Yes	34.81	105	5	OQ221555	https://www.ncbi.nlm.nih.gov/nuccore/OQ221555
ΦCrAssBcn21	97804	Yes	35.18	104	1	OQ221556	https://www.ncbi.nlm.nih.gov/nuccore/OQ221556
ΦCrAssBcn22*	98637	Yes	35.09	105	4	OQ221557	https://www.ncbi.nlm.nih.gov/nuccore/OQ221557
ΦCrAssBcn23	98396	Yes	34.29	101	4	OQ221558	https://www.ncbi.nlm.nih.gov/nuccore/OQ221558
ΦCrAssBcn24	97685	Yes	35.08	103	1	OQ221559	https://www.ncbi.nlm.nih.gov/nuccore/OQ221559
ΦCrAssBcn25	75754	No	35.18	70	-	OQ221560	https://www.ncbi.nlm.nih.gov/nuccore/OQ221560

(*) Species reference genome.

Supplementary Table 4. Complete non-redundant crAss-like phage genomes of family *Steigviridae* used in this study (https://ictv.global/taxonomy).

Subfamily	Genus	Species	GenBank code	Description	
Asinivirinae	*Akihdevirus*	*Akihdevirus balticus*	KC821624	*Cellulophaga* phage phi14:2	dsDNA
Asinivirinae	*Kahnovirus*	*Kahnovirus copri*	MZ130483	uncultured phage cr44_1	dsDNA
Asinivirinae	*Kahnovirus*	*Kahnovirus oralis*	MT774390	uncultured phage cr85_1	dsDNA
Asinivirinae	*Kehishuvirus*	*Kehishuvirus splanchnicus*	MT774388	uncultured phage cr112_1	dsDNA
Asinivirinae	*Kehishuvirus*	*Kehishuvirus primarius*	MH675552	*Bacteroides* phage CrAss001	dsDNA
Asinivirinae	*Kolpuevirus*	*Kolpuevirus coli*	MZ130478	uncultured phage cr151_1	dsDNA
Asinivirinae	*Kolpuevirus*	*Kolpuevirus hominis*	MT774391	uncultured phage cr126_1	dsDNA
Asinivirinae	*Lahndsivirus*	*Lahndsivirus rarus*	MT774387	uncultured phage cr111_1	dsDNA
Asinivirinae	*Lebriduvirus*	*Lebriduvirus gastrointestinalis*	MZ130485	uncultured phage cr18_1	dsDNA
Asinivirinae	*Mahlunavirus*	*Mahlunavirus rarus*	MT774392	uncultured phage cr128_1	dsDNA
Asinivirinae	*Mahstovirus*	*Mahstovirus faecalis*	MT774378	uncultured phage cr106_1	dsDNA
Asinivirinae	*Pamirivirus*	*Pamirivirus faecium*	MT774389	uncultured phage cr116_1	dsDNA
Asinivirinae	*Paundivirus*	*Paundivirus hollandii*	KJ003983	IAS virus	dsDNA
Asinivirinae	*Pipoluvirus*	*Pipoluvirus rarus*	MT774385	uncultured phage cr108_1	dsDNA
Asinivirinae	*Wulfhauvirus*	*Wulfhauvirus bangladeshii*	MT074136	*Bacteroides* phage DAC15	dsDNA

Supplementary Table 5.- Proteins absent in some of the crAssBcn genomes. The presence (white) or absence (grey) of the ORFs in the 24 crAssBcn phages genomes are presented in comparison with the ORFs in the ΦCrAss001. The protein encoded by each ORF has been indicated according with the most recent annotation (January 2023) of the ΦCrAss001 genome.

CrAss001_ORF Nº protein	% ORF present	%ORF absent	1	2	3	4	5	6	7	8	9	10	11	12	13	14	15	16	17	18	19	20	21	22	23	24
CrAss001_18_endonuclease	0	100																								
CrAss001_62_hypothetical protein	8	92																								
CrAss001_26_tail fiber protein	8	92																								
CrAss001_2_hypothetical protein	12	88																								
CrAss001_41_endonuclease	12	88																								
CrAss001_92_hypothetical protein	24	76																								
CrAss001_37_endonuclease	28	72																								
CrAss001_99_HNH endonuclease	28	72																								
CrAss001_8_putative HNH endonuclease	28	72																								
CrAss001_102_CcmD family protein	36	64																								
CrAss001_104_transcriptional regulator	36	64																								
CrAss001_98_hypothetical protein	36	64																								
CrAss001_93_hypothetical protein	40	60																								
CrAss001_23_tail fiber protein	52	48																								
CrAss001_24_putative C-type lectin	60	40																								
CrAss001_25_tail fiber protein	64	36																								
CrAss001_58_zinc-ribbon protein	68	32																								
CrAss001_91_SPFH-domain	72	28																								
CrAss001_61_polynucleotide kinase	80	20																								
CrAss001_90_hypothetical protein	80	20																								
CrAss001_96_protein of unknown function	76	24																								
CrAss001_83_ABC-type cobalt transporter	84	16																								
CrAss001_56_His-Xaa-Ser system radical SAM maturase HxsC-like protein	84	16																								
CrAss001_48_shock protein C	84	16																								
CrAss001_101_hypothetical protein	92	8																								
CrAss001_103_hypothetical protein	92	8																								

Supplementary references

1. Puig, A., Queralt, N., Jofre, J., and Araujo, R. (1999). Diversity of Bacteroides fragilis strains in their capacity to recover phages from human and animal wastes and from fecally polluted wastewater. Appl Environ Microbiol *65*, 1772–1776.

2. Tartera, C., and Jofre, J. (1987). Bacteriophages active against Bacteroides fragilis in sewage-polluted waters. Appl Environ Microbiol *53*, 1632–1637.

3. Ebdon, J., Muniesa, M., and Taylor, H. (2007). The application of a recently isolated strain of Bacteroides (GB-124) to identify human sources of faecal pollution in a temperate river catchment. Water Res *41*, 3683–3690.

4. Ogilvie, L.A., Caplin, J., Dedi, C., Diston, D., Cheek, E., Bowler, L., Taylor, H., Ebdon, J., and Jones, B. v (2012). Comparative (meta)genomic analysis and ecological profiling of human gut-specific bacteriophage φB124-14. PLoS One *7*, e35053. 10.1371/journal.pone.0035053.

5. Sperry, J.F., Appleman, M.D., and Wilkins, T.D. (1977). Requirement of heme for growth of Bacteroides fragilis. Appl Environ Microbiol *34*, 386. 10.1128/AEM.34.4.386-390.1977.

6. Rashtchian, A., Dubes, G.R., and Booth, S.J. (1982). Tetracycline-inducible transfer of tetracycline resistance in Bacteroides fragilis in the absence of detectable plasmid DNA. J Bacteriol *150*, 141. 10.1128/JB.150.1.141-147.1982.

7. Wright, D.P., Knight, C.G., Parkar, S.G., Christie, D.L., and Roberton, A.M. (2000). Cloning of a Mucin-Desulfating Sulfatase Gene from Prevotella Strain RS2 and Its Expression Using a Bacteroides Recombinant System. J Bacteriol *182*, 3002. 10.1128/JB.182.11.3002-3007.2000.

8. Bergan, T., and Hovig, B. (1968). A new species, Sphaerophorus intermedius, isolated from empyema. Acta Pathol Microbiol Scand *74*, 421–430. 10.1111/J.1699-0463.1968.TB03496.X.

9. Privitera, F., Dublanchet, A., and Sebald, M. (1979). Transfer of multiple antibiotic resistance between subspecies of Bacteroides fragilis. J Infect Dis *139*, 97–101. 10.1093/INFDIS/139.1.97.

10. Gómez-Doñate, M., Payán, A., Cortés, I., Blanch, A.R., Lucena, F., Jofre, J., and Muniesa, M. (2011). Isolation of bacteriophage host strains of Bacteroides species suitable for tracking sources of animal faecal pollution in water. Environ Microbiol *13*. 10.1111/j.1462-2920.2011.02474.x.

11. Coyne, M.J., Tzianabos, A.O., Mallory, B.C., Carey, V.J., Kasper, D.L., and Comstock, L.E. (2001). Polysaccharide Biosynthesis Locus Required

for Virulence of Bacteroides fragilis. Infect Immun *69*, 4342. 10.1128/IAI.69.7.4342-4350.2001.

12. Payan, A., Ebdon, J., Taylor, H., Gantzer, C., Ottoson, J., Papageorgiou, G.T., Blanch, A.R., Lucena, F., Jofre, J., and Muniesa, M. (2005). Method for isolation of Bacteroides bacteriophage host strains suitable for tracking sources of fecal pollution in water. Appl Environ Microbiol *71*, 5659–5662. 10.1128/AEM.71.9.5659-5662.2005.

13. Sutter, V.L., Emmerman, J., Randall, E., Zabransky, R.J., and Birk, R.J. (1985). Establishment of MICs of moxalactam for control and reference anaerobic organisms in agar dilution and microdilution techniques. Antimicrob Agents Chemother *27*, 424. 10.1128/AAC.27.3.424.

14. Eggerth, A.H., and Gagnon, B.H. (1933). The Bacteroides of Human Feces. J Bacteriol *25*, 389. 10.1128/JB.25.4.389-413.1933.
