# Supplementary material for: Characterization of crAss-like phage isolates highlights Crassvirales genetic heterogeneity and worldwide distribution
Source: Nat Commun. 2023 Jul 18;14:4295. doi: 10.1038/s41467-023-40098-z (PMC10354031; doi:10.1038/s41467-023-40098-z)
Supplement: Supplementary file 3 — Description of Additional Supplementary Files [file 41467_2023_40098_MOESM3_ESM.docx]

**Description of Additional Supplementary Files**

**File Name: Supplementary Data 1
Description:** Functional annotation of the 25 crAssBcn phages and phage ɸCrAss001. Sheet 1 (annotation platforms) shows the functional annotation was performed using InterPro member databases (SMART, PFAM, CCED and TIGRFAM), and the different results are presented. The second sheet (annotation %AAI with ɸCrAss001) presents the AAI of each gene compared with the curated and revised annotation of ɸCrAss001.

**File Name: Supplementary Data 2
Description:** Variable ORFs in the group of 24 crAssBcn phages. Reciprocal best match of each crAssBcn species based on AAI% when comparing the ORFs of the representative phage of each species (I-VI) against the representative phages of the other six species.

**File Name: Supplementary Data 3
Description:** Origin of published datasets and genes used in this study. Modification of Gregory et al (2020). In the first sheet (Metagenomes) green those showing positive hits and in orange those showing no hits. The other sheets show the genes in databases used for comparison with the seven ORFs showing the highest variability.
