# Supplementary material for: Characterization of crAss-like phage isolates highlights Crassvirales genetic heterogeneity and worldwide distribution
Source: Nat Commun. 2023 Jul 18;14:4295. doi: 10.1038/s41467-023-40098-z (PMC10354031; doi:10.1038/s41467-023-40098-z)
Supplement: Supplementary file 7 — Reporting Summary [file 41467_2023_40098_MOESM7_ESM.pdf]

nature portfolio

Corresponding author(s): Maite Muniesa

Last updated by author(s): Jul 5, 2023

Reporting Summary

Nature Portfolio wishes to improve the reproducibility of the work that we publish. This form provides structure for consistency and transparency in reporting. For further information on Nature Portfolio policies, see our Editorial Policies and the Editorial Policy Checklist.

Statistics

For all statistical analyses, confirm that the following items are present in the figure legend, table legend, main text, or Methods section.

n/a	Confirmed	
☐	☒	The exact sample size (*n*) for each experimental group/condition, given as a discrete number and unit of measurement
☐	☒	A statement on whether measurements were taken from distinct samples or whether the same sample was measured repeatedly
☐	☒	The statistical test(s) used AND whether they are one- or two-sided<br>*Only common tests should be described solely by name; describe more complex techniques in the Methods section.*
☒	☐	A description of all covariates tested
☒	☐	A description of any assumptions or corrections, such as tests of normality and adjustment for multiple comparisons
☐	☒	A full description of the statistical parameters including central tendency (e.g. means) or other basic estimates (e.g. regression coefficient) AND variation (e.g. standard deviation) or associated estimates of uncertainty (e.g. confidence intervals)
☐	☒	For null hypothesis testing, the test statistic (e.g. *F*, *t*, *r*) with confidence intervals, effect sizes, degrees of freedom and *P* value noted<br>*Give P values as exact values whenever suitable.*
☒	☐	For Bayesian analysis, information on the choice of priors and Markov chain Monte Carlo settings
☒	☐	For hierarchical and complex designs, identification of the appropriate level for tests and full reporting of outcomes
☒	☐	Estimates of effect sizes (e.g. Cohen's *d*, Pearson's *r*), indicating how they were calculated

Our web collection on statistics for biologists contains articles on many of the points above.

Software and code

Policy information about availability of computer code

Data collection	Trimmomatic 0.36 and FastQC v0.11.9 (code https://github.com/s-andrews/FastQC/issues)
Data analysis	fq2fa from IDBA v 1.1.3 repository, Spades v3.13.0, BLAST 2.9.0+, Viridic v1.1, Viptree (https://www.genome.jp/viptree/EVG2017) , Prodigal v2.6.3 , Interproscan v. 5.47-82.0 , enveomics collection (besthit by BlastTab.besthitsorter.pl, reciprocal best match by rbm.rb, recruitment plot file by BlastTab.catsbj.pl , enve.recplot2 from enveomic by R and sequencing depth by BlastTab.seqdepth.pl), (enveomic collection code https://github.com/lmrodriguezr/enveomics.git), heatmapper (code https://github.com/WishartLab/heatmapper) , Plotly by R (https://chart-studio.plotly.com). Muscle (MUltiple Sequence Comparison by Log- Expectation) (https://www.ebi.ac.uk/Tools/msa/muscle/), Viptree (https://www.genome.jp/viptree/), IQ-TREE (http://www.iqtree.org/) and Genious Prime version 2023.1.1. GraphPad Prism 9.

For manuscripts utilizing custom algorithms or software that are central to the research but not yet described in published literature, software must be made available to editors and reviewers. We strongly encourage code deposition in a community repository (e.g. GitHub). See the Nature Portfolio guidelines for submitting code & software for further information.

Data

Policy information about availability of data

All manuscripts must include a data availability statement. This statement should provide the following information, where applicable:

- Accession codes, unique identifiers, or web links for publicly available datasets
- A description of any restrictions on data availability
- For clinical datasets or third party data, please ensure that the statement adheres to our policy

Data availability.- The crAssBcn phages genomes data generated in that support the findings of this study have been deposited in GenBank" with the GenBank accession codes available in Supplementary Table 3 and is publicly available. Other supporting information data generated in this study (tree gene sequences, tree alignments and values) can be found in https://data.cyverse.org/dav-anon/iplant/home/lolesramosub/Ramos-Barbero%2C%20MD%2C%20G%C3%B3mez-G%C3%B3mez%2C%20C.%20%2C%20Sala%2C%20L%20%28...%29%26%20Muniesa%2C%20M.%202023%20Supporting%20information/Ramos-Barbero%202023%20Supporting%20information.rar. The following databases were used: Pfam (http://pfam.xfam.org/), SMART (http://smart.embl-heidelberg.de/) TIGRFAMs (https://www.jcvi.org/research/tigrfams) and CDD (https://www.ncbi.nlm.nih.gov/Structure/cdd/cdd.shtml). Database of metagenomes in Figure 6 is available in Supplementary Data 3. Source data for supplementary figure 2 are provided in the Source Data file. Source data for supplementary figure 6 are provided in Supplementary Data 2. The authors declare that all other data supporting the findings of this study are available within the paper and its supplementary files.

Human research participants

Policy information about studies involving human research participants and Sex and Gender in Research.

Reporting on sex and gender	Not applicable
Population characteristics	Not applicable
Recruitment	Not applicable
Ethics oversight	Not applicable

Note that full information on the approval of the study protocol must also be provided in the manuscript.

Field-specific reporting

Please select the one below that is the best fit for your research. If you are not sure, read the appropriate sections before making your selection.

☒ Life sciences ☐ Behavioural & social sciences ☐ Ecological, evolutionary & environmental sciences

For a reference copy of the document with all sections, see nature.com/documents/nr-reporting-summary-flat.pdf

Life sciences study design

All studies must disclose on these points even when the disclosure is negative.

Sample size	Sample size is not applicable in our study. In the figures, the number of phages used were 25, that corresponded to all the phages that we were able to isolate or to their representatives for each species detected. The number of sewage samples used were the number of samples required to allow the isolation of these 25 phages, since they were sufficient for the goal of the study, that was to isolate a group of new phages from our geographical area. When comparing these phages with other phage genomes the number of genomes corresponded to all the non-redundant genomes available in the databases. When using different metagenomes around the world, we generated a database including all the metagenomes of sufficient quality available in the databases.
Data exclusions	No data was excluded
Replication	All experiments were repeated in triplicate and are reproducible. Bioinformatic analysis were performed in duplicate and are reproducible.
Randomization	This is not applicable in our study since no group of individuals were used. When concerning metagenomes available in databases, all were selected for our study
Blinding	Blinding was not relevant to the study since no group of individuals was selected and no results from given individual or group of individuals were obtained that would required blinding.

Reporting for specific materials, systems and methods

We require information from authors about some types of materials, experimental systems and methods used in many studies. Here, indicate whether each material, system or method listed is relevant to your study. If you are not sure if a list item applies to your research, read the appropriate section before selecting a response.

Materials & experimental systems

n/a	Involved in the study
☒	☐ Antibodies
☒	☐ Eukaryotic cell lines
☒	☐ Palaeontology and archaeology
☒	☐ Animals and other organisms
☒	☐ Clinical data
☒	☐ Dual use research of concern

Methods

n/a	Involved in the study
☒	☐ ChIP-seq
☒	☐ Flow cytometry
☒	☐ MRI-based neuroimaging
